# The influence of operation time for hip hemiarthroplasty on complication rates and mortality in patients with femoral neck fracture: a retrospective data analysis: Letter to Editor

**DOI:** 10.1186/s13018-024-04882-x

**Published:** 2024-07-27

**Authors:** Raju Vaishya, Abhishek Vaish

**Affiliations:** Indraprastha Apollo Hospitals, New Delhi, 110076 India

## Abstract

Ramadanov et al.‘s study highlights the importance of minimizing operative time in HHA for femoral neck fractures. Future prospective studies are needed to explore causality and refine strategies for achieving shorter, yet safe, procedures.

Dear editor,

The recent article by Ramadanov et al. in the Journal of Orthopaedic Surgery and Research provides valuable insights into factors affecting patient outcomes after hip hemiarthroplasty (HHA) for femoral neck fractures [1]. The study’s finding that longer operation times are associated with an increased risk of complications and mortality is particularly noteworthy. Identifying a potential cut-off of 86 min for surgical duration offers a helpful benchmark for surgeons.

We believe that there are some limitations to consider when interpreting the results of this study. Due to the retrospective design, establishing a causal relationship between long operation times and adverse outcomes is challenging. Other unmeasured factors, such as surgical complexity or patient comorbidities, might influence both duration and risk. Additionally, the study’s single-center design limits generalizability. The 86-minute cut-off for operation time might not be universally applicable, as different hospitals might have varying baseline efficiencies.

The study doesn’t delve into the specific reasons why longer surgeries might lead to worse outcomes. It would be helpful to understand if certain complication types were more associated with longer surgeries. Strategies for shortening operative times while maintaining safety and effectiveness, such as specific surgical techniques or preoperative planning, could be valuable additions. Furthermore, the 90-day follow-up period might be too short to capture all potential complications. Finally, surgeon fatigue during longer procedures could be a contributing factor to complications, but the study doesn’t account for this possibility.

We feel that addressing these limitations through further research is crucial. Prospective studies with control groups, multicenter data, examining surgeon techniques, and exploring strategies for efficient HHA procedures would provide valuable insights. By addressing these limitations, future research can build on the groundwork laid by Ramadanov et al. and provide more definitive guidance to surgeons performing HHA for femoral neck fractures.

## Data Availability

No datasets were generated or analysed during the current study.

## References

[1] Ramadanov N, Salzmann M, Voss M et al. The influence of operation time for hip hemiarthroplasty on complication rates and mortality in patients with femoral neck fracture: a retrospective data analysis. *J Orthop Surg Res* 19, 311 (2024). 10.1186/s13018-024-04797-7. 10.1186/s13018-024-04797-7 PMC11129483 38802945

