# Correction: The influence of operation time for hip hemiarthroplasty on complication rates and mortality in patients with femoral neck fracture: a retrospective data analysis: Letter to Editor

**DOI:** 10.1186/s13018-024-04977-5

**Published:** 2024-08-17

**Authors:** Raju Vaishya, Abhishek Vaish

**Affiliations:** Indraprastha Apollo Hospitals, New Delhi, 110076 India

**Correction: Journal of Orthopaedic Surgery and Research (2024) 19:438**

10.1186/s13018-024-04882-x

In this article the title was incorrectly given as ‘The influence of operation time for hip hemiarthroplasty on complication rates and mortality in patients with femoral neck fracture: a retrospective data analysis:’ but should have been ‘The influence of operation time for hip hemiarthroplasty on complication rates and mortality in patients with femoral neck fracture: a retrospective data analysis: Letter to Editor’.

The original article has been corrected.

